# The Work of the Factory Nurse

**Published:** 1919-03-29

**Authors:** 


### THE EDITOR'S LETTER-BOX.

[Correspondence on all subjects is invited, but we cannot in any way be responsible for the opinions expressed by our correspondents, who must give their name and address as a guarantee of good faith, but not necessarily for publication. Correspondents are reminded that brevity of style and conciseness of statement greatly facilitate early insertion.]

### THE WORK OF THE FACTORY NURSE.

*To the Editor of* THE HOSPITAL.

SIR,—I am much obliged to Miss Kerr for her answer to my letter *re* Factory Nurse's Salary. It is a very concise statement of the welfare supervisors' point of view, which seems to be that the nurse's position as regards salary, status, and promotion, etc., in factory welfare is to be decided by the Welfare Supervisors' Association (very many of whom are *not* qualified nurses and have only a very superficial knowledge of nursing), and the nurses are to have no voice in the matter.

With regard to the statement that "the nurse is a specialist in one branch only, while the assistant welfare supervisor is a specialist in many," the generally accepted meaning of the term "specialist" is one who devotes his or her entire time to work on a special subject (a working knowledge is the most the majority of us can expect to gain on more than one, especially if we have to combine working for our daily bread along with the pursuits of knowledge).

You will also note that the only promotion offered to the nurse is "to be an assistant welfare supervisor." In other words, if you want to succeed as a factory nurse you must cease to be one and take a step up (?) to the post of assistant welfare supervisor! Does not this create a peculiar situation? It seems to me to warrant the nursing profession taking some steps to define the position of the nurse in factory welfare work from the standpoint of the fully-qualified nurse. I think it proves the need of thoroughly organised effort in conjunction with other nursing services in the task of caring for the health of the nation. The welfare nurse is more than an ambulance attendant—some one to tie up cut fingers and dose fainting, hysterical girls with sal volatile—she has wide opportunities of usefulness if she but keeps her eyes open. For instance, a girl complains of headache; this affords opportunity for looking at the tongue and teeth and for a word in season on the care of the teeth and proper food; or an outburst of hysteria reveals the fact that the girl has been up all night with a sick relative. A very little sympathy brings out the whole story, and often the nurse can suggest means whereby both watcher and sick friend are helped. A "specialist" makes but a poor factory nurse, and I fear that is the type Miss Kerr has in mind.—Yours faithfully,

SHELL FACTORY NURSE.

